# A 12-Words-for-Life-Nurturing Exercise Program as an Alternative Therapy for Cervical Spondylosis: A Randomized Controlled Trial

**DOI:** 10.1155/2014/961418

**Published:** 2014-03-20

**Authors:** Zhijun Hu, Zhanying Tang, Shiwei Wang, Xiulan Ye, Yongjun Wang, Qi Shi, Qiangling Yang, Shaodan Cheng, Min Huang, Yi Dong, Hongjun Gu

**Affiliations:** ^1^Department of Rehabilitation Medicine, Longhua Hospital, Shanghai University of Traditional Chinese Medicine, Shanghai 200032, China; ^2^Institute of Spine, Shanghai University of Traditional Chinese Medicine, 725 South Wanping Road, Xuhui District, Shanghai 200032, China; ^3^Orthopaedics and Traumatology, Central Hospital of Shanghai Jingan District, Shanghai 200040, China; ^4^Orthopaedics and Traumatology, The Bund Street Community Health Service Center Whampoa District of Shanghai City, Shanghai 200001, China; ^5^Orthopaedics and Traumatology, Shanghai Baoshan District Dachang Town Community Health Service Center, Shanghai 200442, China; ^6^Orthopaedics and Traumatology, Shanghai Xuhui District Lingyun Community Health Service Center, Shanghai 200237, China

## Abstract

In this paper, we carried out a randomized controlled clinical trial to explore the effect of 12-words-for-life-nurturing exercise on patients presenting with cervical spondylosis. After exercise intervention, the mean VAS and NDI scores of the patients decreased significantly and the scores of BP, VT, and MH in SF-36 Health Questionnaire were significantly higher. Exercise therapy showed significant effect on relieving pain and improving vitality and mental health. The 12-words-for-life-nurturing exercise may be a potential effective therapy for patients with cervical spondylosis.

## 1. Introduction 

Cervical spondylosis is a disease resulting from degeneration of facet joints in vertebra [1]. It may be associated with paresthesia, muscle weakness in limbs, and severe pain in neck, shoulder, and back [2]. Cervical spondylosis may also contribute to spinal cord dysfunction, vertebrobasilar insufficiency, and even myelopathy, that is, the most severe sequelae [1, 3–5]. Besides, cervical spondylosis is prevalent in population at any age, especially for elder people. With the increasing trend of incidence, cervical spondylosis has been a health concern highlighted globally [6].

Currently, the treatment for cervical spondylosis varies, including surgery, physical therapy, chiropractic manipulative therapy, and osteopathic manipulative treatment [7–9]. Surgical intervention is suitable for patients with severe or progressive neurologic deficits [10]. Physical therapy showed advantage in restoring range of motion, flexibility, and core strengthening. Alternative therapies also showed immeasurable benefit in cervical spondylosis treatments. Although the developed surgical procedures show effect on alleviating the symptoms, most of patients with cervical spondylosis prefer conservative therapies such as electroacupuncture [11] and massage [12].

Exercise as an alternative treatment for cervical spondylosis is popular in China. In this paper, we analyzed the therapeutic effect of specific 12-words-for-life-nurturing exercise on patients presenting with cervical spondylosis and explored whether it could be a potential therapy for cervical spondylosis that will be widely applied worldwide.

## 2. Methods

### 2.1. Patients

There were total 628 patients according with inclusion criteria from 5 clinical research centers including Longhua Hospital affiliated to Shanghai University of Traditional Chinese Medicine, Shanghai Jing'an District Central Hospital, Shanghai Baoshan District Town Community Health Service Centers, Shanghai Xuhui District Lingyun Community Health Service Centers, and Shanghai Huangpu District Community Health Service Center in bund street, between June 2010 and September 2012.

### 2.2. Inclusion Criteria and Exclusion Criteria

The patients aging from 18 to 60 years were involved in this study. The visual analogue scale (VAS) and the neck disability index (NDI) for the patients were less than 60 and 25, respectively. Strict inclusion criteria were applied in this study. Patients with a history of clinical signs such as pain in neck, subscapular, or shoulder, neck stiffness, and muscular tension, with worsening symptoms including weakness, sensory changes, gait instability, and nondermatomal numbness were included. In addition, the diagnosis of the cervical spondylosis was based on the findings of physical examination, X-radiographic testing, computed tomography (CT), magnetic resonance imaging (MRI), and electromyography (EMG). The included patients were not treated with any exercise method and medicine within 2 weeks before this study.

Besides, the pregnancy women were excluded. Additionally, the exclusion criteria also included patients who were diagnosed with severe spinal cord injury and cervical spinal cord severe compression in cervical spondylotic myelopathy according to imageological examination and patients with a history of drug abuse, poor compliance, intemperance, or unstable working environment which were easy to lose for follow-up. Finally, 250 cases were selected, which conformed to the criteria for the diagnosis of cervical spondylosis. Although the traditional surgical procedures showed quick effect on cervical spondylosis, all participators were willing to accept the exercise therapy and had signed informed consent form before treatment.

### 2.3. Groups

The 250 patients were coded in order and the personal data were enclosed in opaque envelopes. Grouping was performed by SAS6.0 software and the grouping information was informed to participators by experimental responsible officer. Total 250 cases were randomly divided into two groups (125 patients per group) including treatment group and wait-list control group. In addition, there were 50 cases in every clinical research centre.

**Figure 1:**
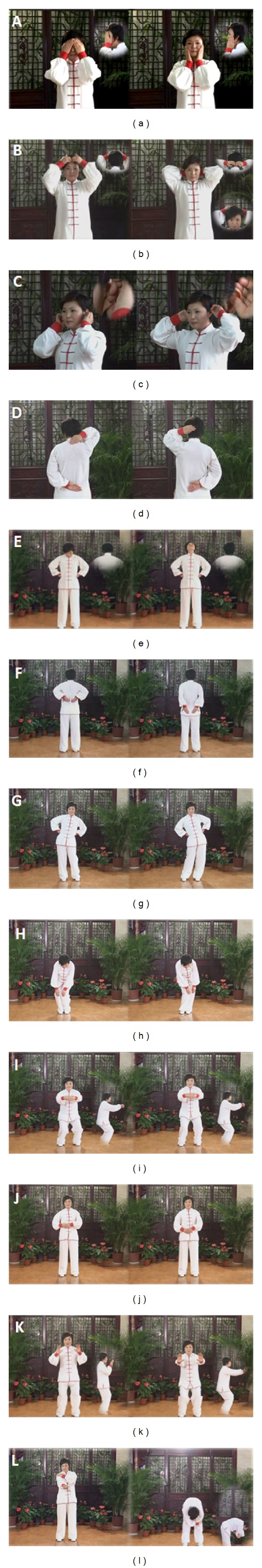


In treatment group, the patients were treated with 12-words-for-life-nurturing exercise including rubbing face by hands (*Xi*) (Figure 1(a)), combing hairs by fingertips (*Shu*) (Figure 1(b)), massaging ears (*Rou*) (Figure 1(c)), rubbing napex (*Cuo*) (Figure 1(d)), loosening neck (*Song*) (Figure 1(e)), pressing waist (*An*) (Figure 1(f)), turning waist (*Zhuan*) (Figure 1(g)), grinding knees (*Mo*) (Figure 1(h)), squatting hip (*Dun*) (Figure 1(i)), rubbing tri-jiao (*Ma*) (Figure 1(j)), deep breathing (*Tu*) (Figure 1(k)), and regulating the limbs (*Tiao*) (Figure 1(l)). In the first 12-week-intervention period, patients were trained for 12-words-for-life-nurturing exercise weekly by responsible officer in each research centre. Every training time remained for around 40 minutes. In addition to training, the patients were required to practice every day for 20 minutes at home until the 24th week. For the patients who did not attend training in time, there would be a supernumerary lesson. If patients missed the supernumerary lessons, they would be excluded.

In wait-list control group, the patients were not treated with any other treatments but were taught with health education concerning cervical spondylosis. The patients were also excluded if they developed more seriously and they must be treated with medicine during the period of investigation.

### 2.4. Follow-Up

At 12 and 24 weeks after training, all the patients underwent follow-up by questionnaire survey and assessment by blind method. The evaluation was primarily the visual analogue scale (VAS) pain assessment before and after exercise treatment. Other evaluation methods contained neck disability index (NDI) assessment and 36-item short-form 36-item Medical Outcome Short Form Health Study Survey (SF-36) questionnaire. NDI assessment was performed according to a previous description [13]. There were 10 items in NDI assessment, such as pain intensity, personal care, driving, headaches, reading, work as well as personal care, lifting, driving, sleeping, and recreational activities. The scores for each items ranged from 0 (no activity limitations) to 5 (major activity limitations). Total score was calculated by summing up scores of each item. Survey questionnaire scores contained 8 main items including role-physical (RP), physical function (PF), general health (GH), bodily pain (BP), social function (SF), vitality (VT), mental health (MH), role-emotional (RE).

### 2.5. Statistical Analysis

The scores of SF-36 Health Questionnaire were normalized. And all of the results were shown as $\overset{-}{x}\pm s$. The data obtained in this paper were analyzed by SPSS 13.0 software. *t*-test and Wilcoxon rank-sum test were performed to compare the data before and after treatment within group. The data corresponded to Gaussian distribution and homogeneity test of variance were compared by variance analysis. And Wilcoxon rank-sum test was carried out for the data which were inconformable to Gaussian distribution.

## 3. Results

### 3.1. Patients

The gender and age of the 250 patients were statistically analyzed. As shown in Table 1, the patients composed of 138 females and 112 males. In treatment group, there were 72 females and 53 males. And in control group, there were 66 females and 59 males. The mean age of patients in treatment group was 44.55 years old, with a range of 22–60. In control group, the mean age of the patients was 45.02 years old ranging from 22 to 60. Among all the patients, 73 patients were diagnosed with cervical spondylotic radiculopathy (CSR) and 19 cases with cervical spondylotic myelopathy (CSM). Total 43 of 73 cases with CSR accepted EMG testing and 7 cases underwent MRI testing. All the CSM patients corresponded to the evidence of MRI testing. There was no significant difference in the baseline information of patients between two groups (*P* > 0.05). Other basic features of the included patients were listed in Table 1.

### 3.2. Groups

At the 12th week, 4 cases in treatment group were excluded for interrupted treatment, and in control group, the conditions of 7 patients worsened. At the 24th week, 9 cases in treatment group and 10 cases in control group were excluded for increased symptom or follow-up difficulties. Finally, there were 112 cases in treatment group and 108 in control group.

### 3.3. VAS Score and NDI Score

Before treatment, the VAS and NDI scores of patients in two groups corresponded to normal distribution. And by *t*-test, there was no statistical difference between treatment group and control group (*P* > 0.05) (Table 1). After treatment, the VAS score and NDI score showed significant differences in treatment group at the 12th week, compared with that at 0 week or 24th week (*P* < 0.01). In contrast, differences were not identified in control group (*P* > 0.05).

At the 12th week after treatment, the difference of VAS score between treatment group and control group was significant (*P* < 0.01). And at the 24th week, VAS score also showed significant difference between the two groups (*P* < 0.01). The results were shown in Table 2. Similar results were found in NDI score assessment (Table 3).

### 3.4. SF-36 Health Questionnaire

Before treatment, the scores of RP, GH, BP, SF, VT, MH, and RE showed in-conformity to Gaussian distribution. By Wilcoxon rank-sum test, the *P* value of all indicators between treatment and control group were more than 0.05. Thus, the physical conditions of the patients between two groups were similar before treatment (Table 1).

After treatment at 12-week and 24 week, the scores of RP, PF, GH, BP, SF, VT, MH, and RE in treatment group all improved significantly compared with those before treatment (*P* < 0.05, Table 4). But in control group, no significant differences were observed (*P* > 0.05). The scores of PF, GH, BP, SF, VT, MH, and RE in treatment group were significantly higher than those in control group, not only at the 12th week but also at the 24th week (*P* < 0.05). Besides, the scores of RP showed similarity at the 12th week between treatment and control group (50.39 ± 18.62 versus 52.78 ± 18.02), while the BR scores showed obviously difference at the 24th week (61.72 ± 20.39 versus 37.30 ± 21.94) (*P* < 0.01).

## 4. Discussion

Cervical spondylosis is a degenerative disease of cervical spine that is susceptible to the individuals with the age more than 40 years [1]. By now, the complement and alternative medicine is popular for the treatment of various diseases all over the world [14]. To the best of our knowledge, this is the first randomized clinical trial to analyze the effect of 12-words-for-life-nurturing exercise compared with no treatment, which was one of traditional Chinese medicine for cervical spondylosis.

Our results suggested that there was no difference in the baseline characters of patients before treatment, including gender, age, VAS, and NDI scores. The VAS and NDI scores of treatment group noticeably decreased compared with those of control group after 12 and 24 week treatment. There was significant difference in VAS and NDI score of treatment group between pretreatment and posttreatment; while in control group, there was no statistical difference. The results of SF-36 health questionnaire also indicated that the normalized BP score was significantly higher than that in control group.

As outlined in previous report, exercise therapy had showed more effect on treating chronic cervical spondylosis than usual care such as analgesics, nonsteroidal anti-inflammatory drugs or muscle relaxants [15, 16]. Qigong as one traditional Chinese exercise showed significant effect on decreasing pain intensity of patients [17]. Pain intensity served as one item addressed in DNI score was proposed to be closely associated with the changes in DNI score [18]. The significant decrease of DNI score in this paper implied that pain intensity of patients reduced after exercise treatment, which showed improvement in disability. Although our results showed significant advantage in VAS score of exercise therapy, the evidence of the benefits for exercise therapy on neck pain treatment was insufficient. It was reported that compared with no treatment, there was no significant effect after 3 months of exercise therapy on elder patients with chronic neck pain [19]. Specific exercise training (dynamic muscle training and relaxation training) appears not to bring about a better outcome of improving neck pain compared with ordinary activity [20]. Additionally, exercise combined with other modality was found to be more effective for chronic cervical spondylosis treatment at three months [21]. Thus 12-words-for-life-nurturing exercise in our study may be more effective on cervical spondylosis, combined with other forms of treatment. Besides, in our work, total of 13 and 17 patients were dropped in treatment group and control group, respectively. And finally, 112 patients of treatment group and 108 ones of control group were included in analysis. It seemed that large scale studies should be conducted to verify the effect of 12-words-for-life-nurturing exercise on VAS score.

Our result also showed that after 12-week and 24-week follow-up, the VT scores and MH scores also showed obvious difference in patients treated with exercise therapy compared with no treatment. At the week 24, the VT score of treatment group was higher than that of control group (66.09 ± 17.58 versus 39.44 ± 21.10) and MH score was 53.13 ± 17.79 versus 39.87 ± 16.29). A previous report showed that mental health and vitality were significantly improved for employees after treated with exercise therapy [22]. Many studies have proved that mental health is closely associated with exercise. Moderate exercise improves mental health by remaining mood and vitality in high levels, which is beneficial for the treatment of mental disorder and the intervention of physical diseases [23]. Tai Chi Quan, another traditional Chinese exercise, has been widely considered to be beneficial for body-mind harmony. In one study, the effect of Tai Chi exercise on mental health was examined. And the results proved that the mental measures of VT and MH were significantly improved for college students with Tai Chi intervention. Tai Chi exercise has been reported to have functions in decreasing blood pressure, changing lipid profiles, and improving both cell-mediated immunity and antibody response [24, 25].

Compared with Tai Chi exercise, the 12-words-for-life-nurturing exercise also has potential in improving body mass index. The exercises of rubbing face by hands (Xi) (Figure 1(a)), combing hairs by fingertips (Shu) (Figure 1(b)), massaging ears (Rou) (Figure 1(c)), and rubbing napex (Cuo) (Figure 1(d)) are considered to dilate blood vessels and promote local blood circulation which may release the pathological factors in neck. Moreover, the 12-words-for-life-nurturing exercises may have benefits in biomechanical balance of cervical region. The disorder of biomechanical balance is one factor leading to the development of cervical spondylosis [26]. The movement form of loosening neck (Song) (Figure 1(e)) has advantage in the neck muscle coordination of movement and maintains the muscle strength balance. Taiji and Qigong exercises are also found to have potential effects on the biomechanical balance [27]. Although the physiological mechanism of the exercise effects on diseases is far from being clear, it seems to indicate that exercise intervention is promising in treating various diseases including cervical spondylosis.

In conclusion, the randomized controlled clinical trial in our study demonstrated that 12-words-for-life-nurturing exercise had promising effect on cervical spondylosis intervention such as relieving pain, improving mental health, and vitality. The 12-words-for-life-nurturing exercise may be a potential alternative therapy for cervical spondylosis. However, whether the exercise can be widely applied worldwide is a problem remaining to be solved and further study should be conducted to determine its effect in the future.

## Figures and Tables

**Table 1:** Baseline information of treatment group and control group.

Classification	Treatment	Control	
	Cases number	125	125
	Sex ratio (M : F)	53 : 72	59 : 66
	Mean age (years old)	44.55 ± 12.42	45.02 ± 12.21
	Mean height (m)	1.70 ± 0.22	1.71 ± 0.25
	Mean weight (kg)	67.35 ± 5.70	68.61 ± 6.51
	BMI (kg/m^2^)	23.31 ± 2.48	23.76 ± 2.21
Types	CSM (%)	10 (8.0)	9 (7.2)
	CSR (%)	35 (28.0)	38 (30.4)
	CS (%)	80 (64.0)	78 (62.4)
Scores	VAS score ($\overset{-}{x}\pm s$)	42.08 ± 5.03	45.02 ± 12.21
	NDI score ($\overset{-}{x}\pm s$)	14.15 ± 2.33	15.12 ± 3.01
SF-36 scores	RP ($\overset{-}{x}\pm s$)	36.71 ± 12.57	37.30 ± 21.93
	PF ($\overset{-}{x}\pm s$)	24.37 ± 10.41	25.39 ± 11.29
	GH ($\overset{-}{x}\pm s$)	39.21 ± 22.40	32.38 ± 22.32
	BP ($\overset{-}{x}\pm s$)	35.00 ± 14.58	36.67 ± 14.14
	SF ($\overset{-}{x}\pm s$)	31.05 ± 6.29	30.75 ± 6.28
	VT ($\overset{-}{x}\pm s$)	44.38 ± 17.56	38.81 ± 19.61
	MH ($\overset{-}{x}\pm s$)	38.06 ± 16.31	39.69 ± 14.82
	RE ($\overset{-}{x}\pm s$)	34.38 ± 25.18	31.75 ± 24.27
	RP ($\overset{-}{x}\pm s$)	36.71 ± 12.57	37.30 ± 21.93
	PF ($\overset{-}{x}\pm s$)	24.37 ± 10.41	25.39 ± 11.29

BMI: body mass index; CSM: cervical spondylotic myelopathy; CSR: Cervical spondylotic radiculopathy; CS: ordinary cervical spondylosis; M : F means male : female; VAS means visual analogue scale; NDI means neck disabled index. RP, PF, GH, BP, SF, VT, MH and RE represent for Role-physical, Physical Function, General Health, Bodily Pain, Social Function, Vitality, Mental Health, Role-Emotional, respectively.

**Table 2:** VAS scores after treatment.

Groups	Pretreatment	Posttreatment	
		12th week	24th week
Treatment	42.08 ± 5.03 (*n* = 125)	33.17 ± 5.94^∗#^ (*n* = 121)	30.07 ± 5.01^∗∗##^ (*n* = 112)
Control	43.18 ± 5.12 (*n* = 125)	42.15 ± 5.48 (*n* = 118)	43.12 ± 6.73 (*n* = 108)

At the 12th week, there were total 121 and 118 patients in treatment group and control group, respectively. At the 24th week, there were total 112 and 108 patients in treatment group and control group, respectively. *P < 0.05, compared with control group; **P < 0.01, compared with control group. ^#^ *P* < 0.05, compared with pretreatment and  ^##^ *P* < 0.01 compared with 12th week.

**Table 3:** NDI scores after treatment.

Groups	Pretreatment	Posttreatment	
		12th week	24th week
Treatment	14.15 ± 2.33 (*n* = 125)	12.97 ± 2.98^∗#^ (*n* = 121)	11.17 ± 3.09^∗∗##^ (*n* = 112)
Control	15.12 ± 3.01 (*n* = 125)	17.25 ± 3.31 (*n* = 118)	17.37 ± 3.34 (*n* = 108)

At the 12th week, there were total 121 and 118 patients in treatment group and control group, respectively. At the 24th week, there were total 112 and 108 patients in treatment group and control group, respectively. *P < 0.05, compared with control group; **P < 0.01, compared with control group. ^#^ *P* < 0.05, compared with pretreatment and  ^##^ *P* < 0.01 compared with 12th week.

**Table 4:** Scores of SF-36 health survey questionnaire after treatment.

Score	12-week follow-up assessment		24-week follow-up assessment	
	Treatment *n* = 121	Control *n* = 118	Treatment group *n* = 112	Control group *n* = 108
RP	50.39 ± 18.6^∗#^	52.78 ± 18.02	61.72 ± 20.39^∗∗##^	52.78 ± 18.02
PF	45.85 ± 14.63^∗#^	24.29 ± 10.39	48.91 ± 14.95^∗∗##^	23.89 ± 11.01
GH	46.64 ± 21.34^∗#^	33.09 ± 621.76	52.66 ± 21.75^∗∗##^	33.33 ± 22.19
BP	48.91 ± 19.61^∗#^	36.03 ± 16.32	53.44 ± 20.95^∗∗##^	41.11 ± 16.86
SF	43.75 ± 10.45^∗#^	18.25 ± 11.84	57.03 ± 14.41^∗∗##^	25.19 ± 11.56
VT	58.82 ± 17.54^∗#^	39.13 ± 19.97	66.09 ± 17.58^∗∗##^	39.44 ± 21.10
MH	47.56 ± 17.02^∗#^	40 ± 15.27	53.13 ± 17.79^∗∗##^	39.87 ± 16.29
RE	51.56 ± 16.72^∗#^	31.75 ± 24.99	71.35 ± 22.90^∗∗##^	36.51 ± 25.19

At the 12th week, there were total 121 and 118 patients in treatment group and control group, respectively. At the 24th week, there were total 112 and 108 patients in treatment group and control group, respectively. RP, PF, GH, BP, SF, VT, MH and RE represent for Role-physical, Physical Function, general health, Bodily Pain, Social Function, Vitality, Mental Health, Role-Emotional, respectively. The scores were calculated as $\overset{-}{x}\pm s$. *P < 0.05, compared with control group; **P < 0.01, compared with control group. ^#^ *P* < 0.05, compared with pretreatment in table1 and ^##^ *P* < 0.01 compared with 12th week.
